# Socio-economic determinants of smoking among Iraqi adults: Data from Non-Communicable Risk Factor STEPS survey 2015

**DOI:** 10.1371/journal.pone.0184989

**Published:** 2017-09-28

**Authors:** Husham J. Abd Al-Badri, Muna Atallah Khaleefah Ali, Ali Abdlkader Ali, Abbas Jabbar Sahib

**Affiliations:** 1 Director of Surveillance Section, Noncommunicable Diseases Prevention and Control Department, Directorate of Public Health, Ministry of Health, Baghdad, Iraq; 2 Director of Noncommunicable Diseases Prevention and Control Department, Directorate of Public Health, Ministry of Health, Baghdad, Iraq; 3 Director of Comprehensive Care for Cardiovascular Diseases and DM Unit, Noncommunicable Diseases Department, Directorate of Public Health, Ministry of Health, Baghdad, Iraq; 4 Director of Tobacco and Substance Control Unit, Mental Health Section, NCD Prevention and Control Department, Directorate of Public Health, Ministry of Health, Baghdad, Iraq; Istituto Di Ricerche Farmacologiche Mario Negri, ITALY

## Abstract

**Aim:**

Highlight the socio-economic determinants of smoking among Iraqi adults aged (18+) years.

**Method:**

The study is derived from Non-Communicable Diseases Risk Factors STEPS survey Iraq 2015. A cross-sectional survey conducted among households from 15 Iraqi governorates. Nainawa, Salahaddin and Al-Anbar were excluded for unstable conditions. Multi-stage cluster sampling technique used to include 4120 Iraqi adults. Interviews started from the first week of November for 20 days using Arabic and Kurdish translated versions of STEPS questionnaire, at last 4071 valid questionnaire forms were gathered.

**Results:**

Among men, smoking rates decline with age, 18–39 years (OR: 1.74; 95%CI: 1.22–2.47) and 40–59 years (OR: 1.69; 95%CI: 1.18–2.44) compared to elderly. They also decline as education level increased, No schooling (OR: 2.74; 95%CI: 1.75–4.31), Less than primary school (OR: 2.46; 95%CI: 1.68–3.62), Primary school (OR: 2.15; 95%CI: 1.51–3.05) and Secondary school (OR: 1.99; 95%CI: 1.33–2.99). They were higher among non-governmental (OR: 1.58; 95%CI: 1.03–2.44) and self-employee (OR: 1.4; 95%CI: 1.06–1.84).

The lowest smoking rates were found among women aged 18–39 years (OR: 0.34; 95%CI: 0.14–0.86). While the highest rates were found among self-employed women (OR: 5.3; 95%CI: 1.12–25.06).

**Conclusions:**

Tobacco smoking was higher among men aged less than 40 years, low educated as well as non-governmental and self-employed people. While it was higher among elderly and self-employed women.

## Introduction

More than five million around the world die as a direct result of tobacco use yearly. This constitutes about one in ten of all deaths among men which exceeded that in women by two times [1]. This may reach up to 8 million smoking related deaths yearly in 2030 if the trend continue [2]. Around 70% of these deaths will occur in developing countries [3].

The most preventable cause of premature death globally could be the usage of tobacco. Cigarettes smoking is blamed for nine out of ten lung cancers, seven in every ten cases of chronic obstructive pulmonary diseases and nearly one fourth of ischemic heart events [1]. Among 4000 types of chemicals detected in the smoke of tobacco, 60 of them are known or suspected to be carcinogens. One hundred million deaths worldwide were due to tobacco use in the 20th century [4]. Passive smoking also causes harmful effects to the people around smokers [3].

In order to reduce this epidemic, the WHO developed the Framework convention for tobacco control FCTC and recommended six demand reduction measures MPOWER to urge the governments to reduce the prevalence of tobacco use and exposure to its smoke [5]. The implementation of which was introduced into the global agenda and presented as a component in the UN Political Declaration on Noncommunicable Diseases and Sustained Developmental Goals [6].

Researches showed an increasing rates of smoking around the globe and that 80% of world smokers reside in low and middle-income countries. In Eastern Mediterranean (EMRO) region, current and past smokers have shown an increased hospitalization rate, healthcare costs and longer hospital stays compared to non-smokers [7].

Previous surveys and studies in Eastern Mediterranean (EMRO) region showed that the prevalence of current smoking ranged from 15.1% in Morocco to 38.5% in Lebanon. Among men it ranged from 20.2% in Saudi Arabia to 62.0% in Syria, while in women it ranged from 0.2% in Morocco to 31.5% in Lebanon [8].

In Iraq the prevalence of current smokers was 21.9% in 2006, and the proportion of smoking among males was six folds higher than females (41.9% vs. 6.9%) [9]. In STEP survey 2015 among Iraqi adults aged (18+ years), the prevalence of current smokers was 20.7%, and the proportion of smoking among males was 20 folds higher than females (38% vs. 1.9%) [10]. Age standardized data showed reduction in the prevalence of tobacco consumption by 0.4% since 2006 survey.

In 2010, reports found that tobacco-caused diseases kill about 10,400 people in Iraq every year [11].

Iraq was able to **M**onitor the trend of tobacco consumption through conduction of several national surveys [9,10,12]. The national tobacco control law was endorsed in 2012 and recently amendments were made to fulfill all of the **MPOWER** requirements in the areas of **P**rotection to cover all facilities of the eight public places by smoking banning, pictorial health **W**arning expanded to occupy 70% of the package, **E**nforcing banning on all forms of direct and indirect advertising, promotion and sponsorship of tobacco products. Also 70% sale tax is **R**aised on all tobacco products by the federal financial budget. Efforts are made to establish tobacco quitting services at selected PHC centers in Baghdad to **O**ffer help to smokers.

Globally tobacco smoking prevalence among men exceeded that in women by five folds; nevertheless, this difference vary considerably across countries. Smoking rates nearly similar in both sexes in high-income countries like Australia, Western Europe and North America countries. While in low- and middle-income countries, smoking rates among men extremely exceeded that for women [13].

Smoking rates also revealed associations with marital status, education level and employment status. Studies showed high rates of smoking among formerly married women and men, low educated as well as precarious and unemployed people especially among men [14].

A study conducted by Center of Diseases Control, United States of America, analyzing the data from 2009 to 2013. The study revealed that highest prevalence of smoking and its related cancers as well as death rates, have shown among low education levels and income counties [15].

The research aims to study the prevalence of smoking among Iraqi adults aged 18 years or more and to assess the socio-economic determinants of smoking among current tobacco smokers.

## Subjects and methods

The study reviewed by scientific comity in Republic of Iraq-Ministry of Health and after approval they submit it to the ethical comity which was headed by Her Excellency Minister of Health.

### Study design

This study was derived from Non-Communicable Diseases Risk Factors STEPS survey Iraq 2015 [10]. A cross-sectional survey conducted among households from fifteen out of eighteen Iraqi governorates. Nainawa, Salahaddin and Al-Anbar were excluded due to the unstable conditions during the time of the survey.

A multi-stage cluster sampling technique had been used to select a representative sample from both sexes of Iraqi adults (18+) years, resided in the urban and rural areas of the selected governorates within 30 days preceding data collection. Temporary residents in Iraq, displaced individuals and those living in institutionalized settings were not included.

### Sample size

The sample size was calculated by the Central Statistical Organization according to the standard formula in STEPS survey report [10]. A total of 412 clusters were randomly selected from the included governorates according to their population proportions, each contain ten households. National rather than a governorate based sample was selected. Primary sampling units (PSUs) were the blocks, which at least consisted of 70 households. One person from each household was randomly selected using KISH table [16] for the interview in the survey with a total sample size of 4120. The sample then weighted to be representative for Iraqi population [10].

### Questionnaire

This study used part of the data that had been collected using STEPS tool version 3 [17]. The questionnaire was tailored and translated into Arabic and Kurdish (For Kurdistan region) by the survey technical committee in collaboration with the WHO for simplification. Central training workshops were carried out in Baghdad and Erbil for the standardized method of utilization of data collection tools. Pilot tests were carried out on accessible samples to estimate the time required to complete the interview and the number of teams needed for data collection.

### Data collection

Data collection started from the first week of November for 20 days. Households had been selected by systematic random sampling technique. Sampling staff from the local statistics directorate updated the sample, localized the selected houses and contacted their residents to schedule the interview with the surveying team. Each surveying team was led by a physician in addition to medical assistant/nurse and laboratory technician.

The interviewers explained the purpose and procedure of the survey then, an informed consent was signed by the selected person before the beginning of the interview.

At the end of data collection 4071 valid questionnaire forms were gathered for STEP 1, with response rate of 98.6%. Part of the data that encounters with socio-demography and smoking status had been analyzed in the current study.

### Dependent variable

**Current smoking**: daily and nondaily smokers within the past 30 days.

### Independent variables

**Socio-demographic:** Age groups, sex, marital status, residence, education level, occupation and monthly per-capita income quintiles.

**Age groups:** The age was classified into three categories (18–39, 40–59, 60+) proportional with sample size in the survey and depending on the main programs at the Non-communicable Diseases Prevention and Control department.

**Monthly per-capita income quintiles:** The monthly income of the interviewed family was divided on the total number of house resident within 30 days before survey. Per-capita income converted to United States dollars (1 US$ = 1200 ID) to be clear for foreign audience, then it was sorted from the lowest to highest and divided into five quintiles (**Quintile 1**: very low <43 US$, **Quintile 2**: very low = 43–73 US$, **Quintile 3**: moderate = 74–107 US$, **Quintile 4**: high = 108–167 US$, **Quintile 5**: very high>167 US$).

### Statistical analysis

EPI-INFO statistical software (Release 2000, version 3.5.4, CDC), had been used for statistical analyses. Post-stratification (according to residence), weighting for generalization of prevalence results on the Iraqi population in the selected governorate. Data had been standardized to the Iraqi adults (18+ years) according to the results of listing and numbering operation done in 2009. While data weighting was not used for analysis of data relations in the current study to prevent over-estimation. The application of sample weights on regression coefficients still not very clear, as well they make the evaluation of regression’s standard errors very difficult in evaluation [18]. Average of age and standard deviation were used for description. Age was also classified and represented by frequency and percentage with other categorical variables. Univariate and multivariate logistic regression models were used to assess the associations between dependent and independent variables. Factors with significant crude odds ratio(s) in Univariate analysis were included in Multivariate logistic regression model and adjusted with each other to calculate the adjusted odds ratios with 95% confidence interval. Odds were regarded significant if its 95% confidence interval did not cross the value of one.

## Results

The current study included 4071 Iraqi adults (18+ years), representing the respondents for STEP 1 of the National STEPS Survey 2015. The main socio-demographic characteristics, according to sex are shown in Table 1.

**Table 1:** Socio-demographic features of Iraqi adults (18+ yrs.), according to their sex 2015.

*Characters*	*Men* *No*. *(%)*	*Women* *No*. *(%)*	*Both sexes* *No*. *(%)*
**Age groups**			
18–39 yrs.	737 (45.8%)	1252 (50.9%)	1989 (48.9%)
40–59 yrs.	574 (35.7%)	843 (34.2%)	1417 (34.8%)
60 + yrs.	298 (18.5%)	367 (14.9%)	665 (16.3%)
**Total**	**1609 (100%)**	**2462 (100%)**	**4071 (100%)**
**Residence**			
Urban	1256 (78.1%)	1945 (79%)	3201 (78.6%)
Rural	353 (21.9%)	517 (21%)	870 (21.4%)
**Total**	**1609 (100%)**	**2462 (100%)**	**4071 (100%)**
**Marital status**			
Single	287 (17.8%)	275 (11.2%)	562 (13.8%)
Married	1272 (79.1%)	1776 (72.1%)	3048 (74.9%)
Previously married	50 (3.1%)	411 (16.7%)	461 (11.3%)
**Total**	**1609 (100%)**	**2462 (100%)**	**4071 (100%)**
**Education level completed**			
No formal schooling	178 (11.2%)	671 (27.3%)	849 (21%)
Less than primary school	295 (18.5%)	620 (25.3%)	915 (22.6%)
Primary school	455 (28.5%)	577 (23.5%)	1032 (25.5%)
Secondary school	215 (13.5%)	228 (9.3%)	443 (10.9%)
High school	192 (12%)	155 (6.3%)	347 (8.6%)
University/higher education	260 (16.3%)	203 (8.3%)	463 (11.4%)
**Total (22 missing)**	**1595 (100%)**	**2454 (100.1%)**	**4049 (100.1%)**
**Occupation**			
Government employee	408 (26.2%)	113 (4.6%)	521 (13.1%)
Non-Gov. employee	111 (7.1%)	23 (0.9%)	134 (3.4%)
Self-employed	465 (29.8%)	20 (0.8%)	485 (12.2%)
Unpaid works	575 (36.9%)	2276 (93.6%)	2851 (71.4%)
**Total (80 missing)**	**1559 (100%)**	**2432 (99.9%)**	**3991 (100.1%)**
**Per capita income / month (quintiles)**			
Very low (<43 US$)	299 (20.6%)	427 (20%)	726 (20.3%)
Low (43–73 US$)	272 (18.8%)	432 (20.2%)	704 (19.6%)
Moderate (74–107 US$)	271 (18.7%)	438 (20.5%)	709 (19.8%)
High (108–167 US$)	328 (22.6%)	470 (22%)	798 (22.3%)
Very high (>167 US$)	279 (19.3%)	369 (17.3%)	648 (18.1%)
**Total (486 missing)**	**1449 (100%)**	**2136 (100%)**	**3585 (100.1%)**

US$ United States Dollars

The un-weighted average of age of the sample was 41.8 years (± 15.7), about half of them were younger than 40 years. Men were generally older with average age of 42.8 years (± 16.5) compared to 41.1 years (± 15.2) for women.

Women constituted 60.5% of the study sample. The majority of the selected sample were from urban areas. Around three fourths of them were married.

Table 1, also showed that about two thirds of the studied sample did not complete the secondary school.

The highest percentage of the participants were unpaid for their work, especially among women where the vast majority were housewives. Around two thirds of the studied sample were classified as moderate to very low monthly per-capita income (≥107 US$).

STEPS survey Iraq (2015) showed that two out of ten Iraqi adults were current smokers, and the vast majority of those (95%) were daily smokers. The prevalence of current smoking was 38% among men, while it was 1.9% among women. The un-weighted number of current smokers were 656, of them 595 were men and 61 women. The majority used manufactured cigarettes with an average of 24 cigarettes/day followed by shisha use. Men reported initiation of smoking at a younger age than women, details showed in Table 2

**Table 2:** Smoking parameters among Iraqi adults (18+ yrs.), according to STEP survey 2015.

	*Men*	*Women*	*Both Sexes*
**Smoking status, No. (Weighted %)**			
Current smoker (daily)	567 (36.12)	58 (1.76)	625 (19.6)
Current smoker (non-daily)	28 (1.91)	3 (0.17)	31 (1.07)
Former smoker	250 (11.67)	72 (2.25)	322 (7.14)
Never smoked	762 (50.31)	2329 (95.83)	3091 (72.2)
**Smoked products, No. (Weighted %)**			
Manufactured cigarettes	483 (79.17)	55 (79.17)	538 (79.17)
Shisha	39 (10.79)	0 (0)	39 (10.31)
Cigar	1 (0.14)	0 (0)	1 (0.13)
No answer	72 (9.9)	6 (20.83)	78 (10.39)
**Mean Age Started Smoking Yrs. (95%CI)**	18.9 (18.4–19.4)	24.7	19.1 (18.6–19.7)
**Mean amount of Tobacco Used by Daily**			
Manufactured cigarettes No. (95%CI)	23.7 (22.3–25.1)	19.2	23.7 (22.3–25.1)
Shisha No. of sessions*	1.7	0	1.7

CI: Confidence interval;

*Session of shisha = 60 minutes

The results showed that; smoking significantly associated with participants' age and occupation in both sexes. It also associated with education level of men but it did not show similar relation with that of women.

Smoking also associated with the marital status and per capita monthly income of women, which did not found with those of men.

Smoking among men and women showed no significant associations with their residence, details in Table 3.

**Table 3:** Univariate analysis of current smoking determinants among Iraqi adults (18+ yrs.), 2015.

*Characters*	*Current smokers/ Total men (%)*	*Crude* *OR (95%CI)*	*Current smokers/ Total women (%)*	*Crude* *OR (95%CI)*
**Age groups**				
18–39 yrs.	294/737 (39.9%)	**2.04 (1.51–2.76)**	13/1252 (1.0%)	**0.22 (0.10–0.45)**
40–59 yrs.	228/574 (39.7%)	**2.02 (1.48–2.76)**	31/843 (3.7%)	**0.79 (0.43–1.44)**
60 + yrs.	73/297 (24.6%)	1 (Reference)	17/367 (4.6%)	1 (Reference)
**Residence**				
Urban	466/789 (37.1%)	1.03 (0.80–1.3)	48/1897 (2.5%)	0.98 (0.53–1.83)
Rural	129/224 (36.5%)	1 (Reference)	13/504 (2.5%)	1 (Reference)
**Marital status**				
Single	110/287 (38.3%)	0.79 (0.43–1.45)	4/275 (1.5%)	**0.31 (0.10–0.91)**
Married	463/1271 (36.4%)	0.73 (0.41–1.29)	38/1776 (2.1%)	**0.45 (0.26–0.79)**
Previously married	22/50 (44.0%)	1 (Reference)	19/411 (4.6%)	1 (Reference)
**Education level completed**				
No formal schooling	67/178 (37.6%)	**1.84 (1.22–2.78)**	26/671 (3.9%)	2.18 (0.51–9.30)
Less than primary school	129/295 (43.7%)	**2.37 (1.65–3.41)**	12/620 (1.9%)	1.07 (0.24–4.83)
Primary school	191/455 (42.0%)	**2.20 (1.57–3.09)**	14/577 (2.4%)	1.34 (0.30–5.99)
Secondary school	84/215 (39.1%)	**1.95 (1.32–2.90)**	4/228 (1.8%)	0.96 (0.17–5.35)
High school	59/192 (30.7%)	1.35 (0.89–2.05)	1/155 (0.6%)	0.64 (0.11–3.90)
University/higher education	64/259 (24.7%)	1 (Reference)	4/203 (2.0%)	1 (Reference)
**Occupation**				
Government employee	153/408 (37.5%)	**1.32 (1.01–1.73)**	3/113 (2.7%)	1.12 (0.35–3.65)
Non-Gov. employee.	49/111 (44.1%)	**1.74 (1.15–2.64)**	1/23 (4.3%)	1.87 (0.25–14.13)
Self-employee.	205/465 (44.1%)	**1.74 (1.35–2.24)**	2/20 (10.0%)	**4.57 (1.04–20.20)**
Unpaid works	179/574 (31.2%)	1 (Reference)	54/2276 (2.4%)	1 (Reference)
**Per capita income / month**				
Very low (<43 US$)	126/299 (42.1%)	1.24 (0.89–1.73)	14/427 (3.3%)	0.80 (0.38–1.68)
Low (43–73 US$)	98/272 (36.0%)	0.96 (0.68–1.35)	5/432 (1.2%)	**0.28 (0.10–0.77)**
Moderate (74–107 US$)	103/271 (38.0%)	1.04 (0.74–1.47)	10/438 (2.3%)	0.55 (0.25–1.24)
High (108–167 US$)	110/328 (33.5%)	0.86 (0.61–1.20)	8/470 (1.7%)	**0.41 (0.17–0.98)**
Very high (>167 US$)	103/278 (37.1%)	1 (Reference)	15/369 (4.1%)	1 (Reference)

OR = Odds Ratio, Bold values were significant (<0.05 by Univariate analysis),

The adjusted odds of smoking significantly decreased with men age. In reverse, they were significantly lower among younger women. It also showed a significant decline as education level of men increased in general. High school graduates precluded from this significant relation. Non-governmental and self-employees men had significantly higher odds of being smoker. The highest odd of smoking was among self-employed women rather than other occupational categories.

Marital status and monthly per-capita income of women found to be confounders with unreal relations with smoking, Table 4.

**Table 4:** Multivariate logistic regression analysis of current smoking determinants among Iraqi adults (18+ yrs.), 2015.

*Characters*	*Men Adjusted OR (95%CI)*	*Women Adjusted OR (95%CI)*
**Age groups**		
18–39 yrs.	**1.74 (1.22–2.47)**	**0.34 (0.14–0.86)**
40–59 yrs.	**1.69 (1.18–2.44)**	1.09 (0.52–2.27)
60 + yrs.	1 (Reference)	1 (Reference)
**Marital status**		
Single		0.31 (0.07–1.48)
Married		0.54 (0.28–1.06)
Previously married		1 (Reference)
**Education level completed**		
No formal schooling	**2.74 (1.75–4.31)**	
Less than primary school	**2.46 (1.68–3.62)**	
Primary school	**2.15 (1.51–3.05)**	
Secondary school	**1.99 (1.33–2.99)**	
High school	1.42 (0.92–2.18)	
University/higher education	1 (Reference)	
**Occupation**		
Government employee	1.34 (0.99–1.79)	0.84 (0.24–2.93)
Non-Gov. employee	**1.58 (1.03–2.44)**	2.71 (0.33–22.42)
Self-employed	**1.40 (1.06–1.84)**	**5.30 (1.12–25.06)**
Unpaid works	1 (Reference)	1 (Reference)
**Per capita income / month**		
Very low (<43 US$)		0.72 (0.33–1.58)
Low (43–73 US$)		0.26 (0.09–0.75)
Moderate (74–107 US$)		0.46 (0.19–1.10)
High (108–167 US$)		0.39 (0.16–0.95)
Very high (>167 US$)		1 (Reference)

OR = Odds Ratio, Bold values were significant (<0.05 by Multivariate logistic analysis)

## Discussion

The prevalence of current smoking in Iraq 2015 was 20.7%. It ranked in the middle zone in comparison to the Eastern Mediterranean region countries that ranged from 11.8% in Iran 2009 [19] to 38.5% in Lebanon 2008. [20] The prevalence of current smoking in other surveys were 16.4% in Qatar [21] and 20.5% in Egypt [22] in 2012. While it was 24.4% in Kuwait 2015 [23]. This was mostly due to similar social and demographic structures of these countries. This had tremendous impact on their habits or the report of such behaviors.

All the surveys revealed that manufactured cigarettes were the most used type of tobacco product among current smokers, as it is the most affordable and easily accessible.

Women constituted 60.5% of the studied sample. Women dominance were seen in surveys from Lebanon [20], and Qatar [21], where women consisted 54.9%, and 57.8% respectively. Surveys in Iraq always show a dominance of women as men had the responsibilities for working outside as well as the continuous wars that recruit the men only.

The present study showed that current smoking was more prevalent among men aged younger than 40 years. Modernization and wide spread of coffee shops that serve. As well, the spread of varieties of smoked types like E-cigarettes, cigars, and several types of cigarettes with affordable prices and proportionally low taxes. Above factors could be the reason of high prevalence of smoking among younger age men. In women smoking was significantly higher among older age groups. A similar picture was in 2006. This was in agreement with the findings of a review of 2007 STEPS survey in Jordan where current smoking was more prevalent among younger men and elderly women [24]. In the survey that had been conducted among 1435 individuals in the North of Iran, smoking was prevalent among middle age group, and in older age women [25]. Another survey from Iran carried out among 1359 women of reproductive age (15–49 years) also showed a high prevalence among older age group (>40 years) [24]. A survey that conducted in Pakistan 2012, included 2644 participant also showed that current smoking was significantly prevalent among middle age group [26].

The recorded low prevalence of smoking among women especially younger age groups might be affected by under-reporting due to social barriers experienced in the region [19–22].

Marital status did not show a real impact on smoking among both men and women. This was also reported in a study among 8045 community based adults from Turkey [27]. Unfortunately, relation of smoking with marriage "if it was real" could be used by healthcare givers as trigger to urge smokers to quit by the effect of their spouses.

Prevalence of smoking was reversibly associated with educational level of men but showed no association with women educational level. This was in agreement with the findings of two studies from Pakistan [26,28]. Protective effect of education from smoking also found by Aryal et al. in a survey based study conducted in Nepal 2013 among 4200 respondents aged 18–69 years [29]. Low educated people had low contact with health education about smoking hazards and more prone for stress in their work that might force them to smoke.

The study also revealed that non-governmental and self-employees had the highest risk for being smoker. This might be due to work stress and competition in addition to limited smoking restrictions at such work places. This relation was confirmed by Ayyagari and Sindelar in a study published at 2010 [30]. Similar findings were reported by Sreeramareddy and Pradhan in a study analyzing smoking determinants from STEPS surveys of 15 Countries around the globe [31]. Unemployed or unpaid categories had the lowest odds for being smokers in all the countries involved, but this relation was not significant among Egyptian women [31]. This might be understood as the vast majority of those people are women (housewives especially) and smoking among them are extremely under-reported for social reasons mostly. Those community layers still need more efforts and health-education about the hazards of such habit even though they had the least smoking percentage. Unemployed or unpaid men are mostly old ages and have chronic diseases so they either quit smoking or non-smoker.

Per-capita monthly income did not show a significant association with smoking status of the participants. Income shown to be a confounding factor among women and the true effect was the employment status as unpaid workers mainly had very low prevalence and may be the lowest monthly income. In addition, very low prices of the cigarettes in Iraq made it affordable to all economic levels with no significant differences. This finding was supported by studies from Iran [25], Pakistan [26] and Turkey [27].

### Conclusions and recommendations

Based on the results, Iraq is ranked in the mid-zone of Eastern Mediterranean regional countries regarding current tobacco smoking. The majority used manufactured cigarettes with the increase of shisha usage. Younger age with low education level as well as self-and/or unemployed persons were the most significant risk factors for current smoking among Iraqi men, while elderly and self-employed women represented the most risky groups among Iraqi women.

Despite the high efforts of Ministry of health and the multi-sectorial steering committee in Iraq, tobacco control interventions confronted serious constraints related to modernization and unstable conditions. Hence, minor reduction of smoking prevalence was achieved since 2006. Reinforcement of multi-sectorial implementation of tobacco demand reduction measures MPOWER is required. Focusing on provision of educational materials for target groups within teaching curriculum, offering tobacco quitting services, and rising of total and retail taxes of cigarettes might be the most effective measure in tobacco control efforts in Iraq.
